# Disproportionately higher unintentional injury mortality among Alaska Native people, 2006–2015

**DOI:** 10.1080/22423982.2017.1422671

**Published:** 2018-01-18

**Authors:** Gretchen Day, Peter Holck, Hillary Strayer, Kathryn Koller, Timothy Thomas

**Affiliations:** ^a^ Clinical & Research Services, Alaska Native Tribal Health Consortium, Anchorage, AK, USA; ^b^ Wellness & Prevention, Alaska Native Tribal Health Consortium, Anchorage, AK, USA

**Keywords:** Alaska Native, Unintentional Injury, Mortality, Poisoning, Urban-Rural

## Abstract

We compared rates of unintentional injury (UI) deaths (total and by injury category) among Alaska Native (AN) people to rates of U.S. White (USW) and Alaska White (AKW) populations during 2006–2015. The mortality data for AN and AKW populations were obtained from Alaska Bureau of Vital Statistics and USW mortality data were obtained from WISQARS, the Center for Disease Control and Prevention online injury data program. AN and AKW rates were age-adjusted to the U.S. 2000 Standard Population and rate ratios (RR) were calculated. AN people had higher age-adjusted total UI mortality than the USW (RR = 2.6) and AKW (RR = 2.3) populations. Poisoning was the leading cause of UI death among AN people (35.9 per 100,000), more than twice that of USW (RR = 2.9) and AKW (RR = 2.5). Even greater disparities were found between AN people and USW for: natural environment (RR = 20.7), transport-other land (RR = 12.4), and drowning/submersion (RR = 9.1). Rates of AN UI were markedly higher than rates for either USW or AKW. Identifying all the ways in which alcohol/drugs contribute to UI deaths would aid in prevention efforts. All transportation deaths should be integrated into one fatality rate to provide more consistent comparisons between groups.

## Introduction

Often referred to as accidents, unintentional injuries (UI) are preventable injuries for which harm either to oneself or others is not intended. UI mortality, comprised of a wide variety of unintentional death categories, is the leading cause of death among persons younger than 45 years in the USA [1]. Among Alaska Native (AN) people, unintentional injuries are also the leading cause of death for persons 45–70 years, ages where chronic disease deaths supplant UI among most of the US population [2,3].

In 2015, an estimated 144,274 AN people represented approximately 18% of the state’s total population [4]. More than a third (38%) of AN people reside in about 200 rural communities, 178 of which are not connected by roads and are separated from each other and regional hospitals by vast stretches of tundra, water, glaciers and mountains. Year-round travel relies on small plane, which is supplemented seasonally by boat, snow machine or all-terrain vehicle. Alaska contains more than 50% of the coastline of the US and has thousands of miles of rivers. Every year, tens of thousands of AN people harvest, process, distribute and consume millions of pounds of wild animals, fish and plants as part of a “subsistence” lifestyle. Subsistence activities necessitate time spent on or near water, or exposed to other natural hazards [5]. Geographic remoteness also impedes medical care access and may play an additional role in UI mortality rates.

This study examines categories of UI mortality among AN people. We compared AN rates with US White (USW) and Alaska White (AKW) rates during 2006–2015 and examined differences in rates between age groups; sexes; and urban, rural and remote areas in Alaska.

## Methods

We obtained death certificate data for all fatal UI involving Alaska residents during 2006–2015 from the State of Alaska Bureau of Vital Statistics. We defined AN people as those for whom the death certificate race code specified Alaska Native, Eskimo or Canadian Eskimo, Indian or Canadian Indian, Aleut, or a mixture including any of these groups, both Hispanic and non-Hispanic. AKW were defined by a race code which specified White, both Hispanic and non-Hispanic. We obtained bridged population estimates for both AN and AKW populations from the State of Alaska Labor and Workforce Development population estimates for the time period (2006–2015) [6,7].

We compared AN mortality rates per 100,000 by age and sex to AKW and USW rates per 100,000 for total UI and for the leading injury categories among AN people based on frequency. UI category definitions (Supplemental Appendix A) [8] and USW data for 2006–2015 were obtained from the US Centers for Disease Control and Prevention’s Web-based Injury Statistics Query and Reporting System (WISQARS) [9]. UI poisoning deaths include deaths by drugs (includes over-the-counter [OTCs], prescription medications and illegal forms), alcohol, gases and vapours and other unspecified chemicals and noxious substances; UI motor vehicle-traffic mortality includes motorcycle, car, truck, bus and heavy vehicle traffic crashes/incidents as well as incidents where pedestrians, pedal cyclists and off-road vehicle occupants are killed by motor vehicles; and UI drowning/submersion mortality includes deaths while in or falling into a bath tub, swimming pool or natural waters. UI natural environment mortality encompass death from exposure to excessive heat or cold, air pressure changes, hunger, thirst, venomous plants and animals, other wildlife encounters, lightning, cataclysmic storms, and movements of the earth, including avalanches. Death by transport – other (land) includes deaths involving all non-traffic land vehicles (e.g. all-terrain vehicles [ATVs], snow machines). To reduce variability produced by a small AN population and increase statistical power, we aggregated deaths for the 10-year period to estimate mortality rates. We then age-adjusted rates to the US 2000 Standard Population for all ages combined. To protect privacy, we do not report specific estimates for categories of UI with fewer than 5 deaths during the time period.

We classified decedents by location of residence at the time of death. A large proportion (38%) of the AN population lives in rural villages or small towns off the road system. Since the US census definition of “urban area” as 50,000 or more people inadequately captures differences in Alaska census places, we designated each census place into 1 of 3 categories (urban, rural, or remote) based on an isolation score developed as part of an Alaska Rural Primary Care Facility Needs Assessment Project [10]. Scores reflect the level of emergency medical services (EMS) offered at each census place and the distance and mode of travel to the nearest hospital (road, air or water). The most urban of the 3 classification levels of census places includes places within 100 road miles of EMS category I or II services. The second level (rural) consists of locations 100–600 miles (161–966 km) by road to EMS category I or II services, and the third level (remote) includes all places requiring air or water travel, or that are more than 600 miles (966 km) by motor vehicle from EMS services. About 60% of AN people live in urban, 10% in rural and 30% in remote locations. In contrast to national definitions, this definition of “urban” location includes towns of 6000 people living hundreds of miles from the road system.

Differences in mortality rates were compared by age group, sex and urban/rural/remote designation using rate ratios based on age-adjusted rates of AN and AKW UI deaths. All statistical analyses were performed using R statistical package [11].

## Results

Of the 37,896 resident deaths occurring in Alaska during 2006–2015, death certificate data were obtained for 3,573 (9.4%) classified as UI. Table 1 provides the average annual age-adjusted UI mortality rates per 100,000 for AN, AKW and USW peoples and rate ratios (RR) for AN/USWs and AN/AKWs. The average annual age-adjusted UI mortality rate for AN people (108.5 per 100,000) was significantly greater than USWs (RR = 2.6, 95% CI 2.4–2.8) and AKWs (RR = 2.3, 95% CI 2.2–2.5). Sex-specific UI mortality rates were also significantly greater among AN people than for USWs (males: RR = 2.5, 95% CI 2.3–2.7; females: RR = 2.7, 95% CI 2.5–3.1) and AKWs (males: RR = 2.3, 95% CI 2.1–2.5; females: RR = 2.5, 95% CI 2.2–2.9).

**Table 1.** Average annual age-adjusted unintentional injury mortality rates per 100,000 for AN people compared to USW and AKW, for leading causes among AN people 2006–2015.

	Male and female combined						Males						Females					
WISQARS ICD10 code groups	AN deaths	AN rate	USW rate	AN/USW rate ratio	AKW rate	AN/AKW rate ratio	AN deaths	AN rate	USW rate	AN/USW rate ratio	AKW rate	AN/AKW rate ratio	AN deaths	AN rate	USW rate	AN/USW rate ratio	AKW rate	AN/AKW rate ratio
All unintentional injury	**1104**	**108.5**	**41.9**	2.6*	**46.5**	2.3*	**722**	**140.0**	**56.6**	2.3*	**61.3**	2.3*	**381**	**76.8**	**28.0**	2.5*	**30.3**	2.5*
(1) Poisoning	380	35.9	12.6	2.9*	14.2	2.5*	209	38.9	16.5	2.4*	18.2	2.1*	171	32.8	8.7	3.8*	9.6	3.4*
(2) Motor vehicle-traffic	150	13.1	11.9	1.1	8.6	1.5*	92	16.1	17.0	0.95	11.6	1.4*	58	9.9	6.9	1.4*	5.5	1.8*
(3) Drowning/submersion	113	10.1	1.1	9.1*	2.4	4.3*	97	17.4	1.7	10.2*	3.7	4.7*	15	2.5	0.5	4.8*	0.9	2.9*
(4) Natural environment	99	9.6	0.5	20.7*	1.1	8.4*	69	12.7	0.7	19.2*	1.8	7.1*	30	6.2	0.3	22.5*	0.4	15.6*
(5) Transport-other (land)	69	6.2	0.5	12.4*	1.2	5.1*	56	10.7	0.8	13.0*	1.8	5.9*	13	2.1	0.2	10.8*	0.5	4.0*

*Significantly different from 1, p< 0.05.

**Figure 1.**
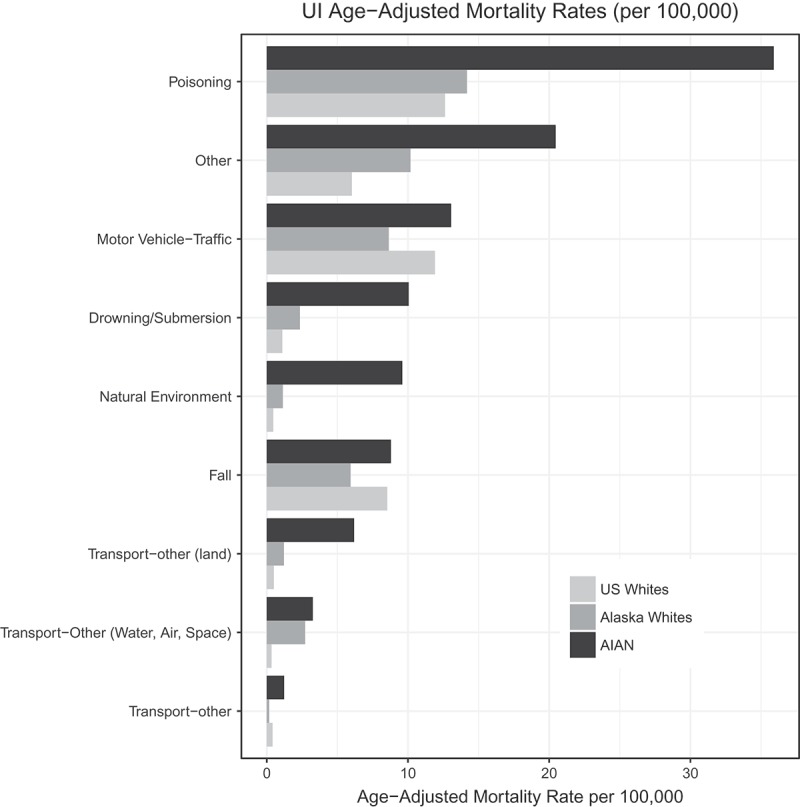
Comparison of age-adjusted rates for leading categories of unintentional injury mortality.

**Figure 2.**
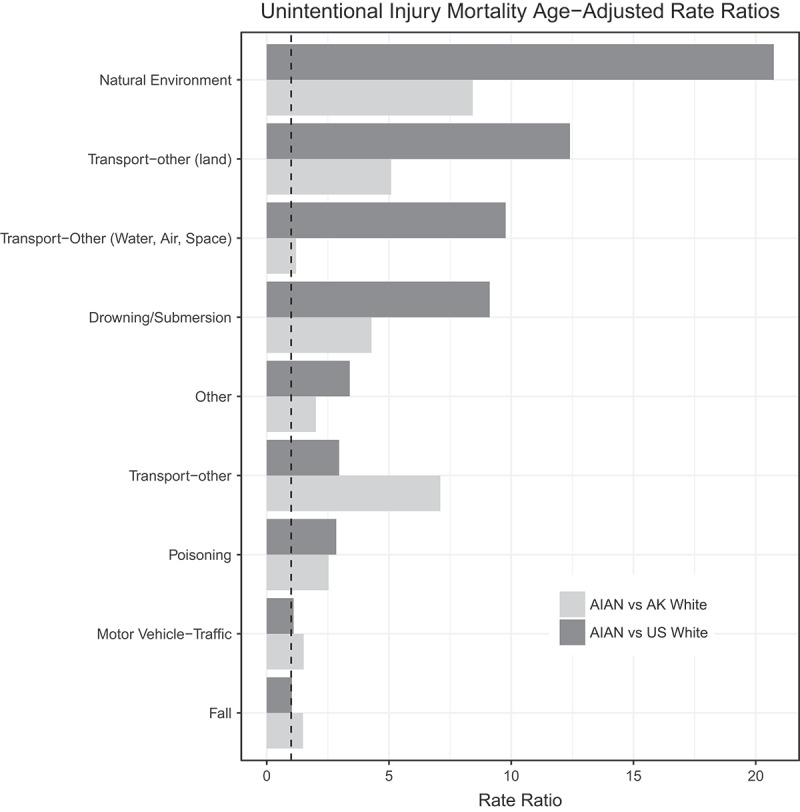
Rate ratios comparing AIAN to Alaska White and US White populations for leading categories of unintentional injury mortality.

The 5 leading UI categories for AN deaths overall were *poisoning, motor vehicle-traffic* crashes/incidents, *drowning/submersion, natural environment* and *transport-other (land*), accounting for 73% (n = 811) of all AN UI mortality. *Falls*, the third leading cause of UI death among USWs and AKWs, was not among the 5 leading causes of overall death in AN people, although rates were higher than USW or AKW (data not shown). However, *drowning/submersion, natural environment* and *transport-other (land)*, being the third, fourth and fifth leading causes of death for AN people, were not among the 5 leading causes of overall death for USWs and AKWs. While AN mortality rates were significantly higher than USW for *poisoning, transport-other (land), drowning/submersion* and *natural environment*, the rates of *motor vehicle-traffic* mortality were similar. AN mortality rates were significantly higher than AKW rates for all leading categories (Table 2). Age adjusted mortality rates by race are also shown in Figure 1, and rate ratios are displayed in Figure 2.

**Table 2.** Top 5 leading causes of unintentional injury mortality for AN people, AKW and USW, 2006–2015.

Rank	WISQARS ICD10 code group	AN rate	Rank	WISQARS ICD10 code group	AKW rate	Rank	WISQARS ICD10 code group	USW rate
	All unintentional injury	**108.5**		All unintentional injury	**46.5**		All unintentional injury	**41.9**
**1**	Poisoning	35.9	**1**	Poisoning	14.2	**1**	Poisoning	12.6
**2**	Motor vehicle-traffic	13.1	**2**	Motor vehicle-traffic	8.6	**2**	Motor vehicle-traffic	11.9
**3**	Drowning/submersion	10.1	**3**	Falls	6.2	**3**	Falls	8.4
**4**	Natural environment	9.6	**4**	Transport-other (water, air, space)	3.0	**4**	Suffocation	1.9
**5**	Transport-other (land)	6.2	**5**	Suffocation	2.9	**5**	Fire/Burn	0.8

AN age-specific death rates for all UI were significantly higher than USW and AKW for every age category overall (more than twice as high in all but the elderly age group) and for age group stratified by sex (Table 3). Comparisons of AN people and USW indicate RR was highest in the 0–19 age group and lowest in the 65+ age group. A similar pattern existed between AN people and AKW. In all the 3 population groups, age-specific rates were higher among males than females.

**Table 3.** Age-specific average annual UI mortality rates per 100,000 for AN people compared to USW and AKW, 2006–2015.

	Male and female combined					Males					Females				
WISQARS ICD10 code groups 0–19 years	AN rate	USW rate	AN/USW rate ratio	AKW rate	AN/AKW rate ratio	AN rate	USW rate	AN/USW rate ratio	AKW rate	AN/AKW rate ratio	AN rate	USW rate	AN/USW rate ratio	AKW rate	AN/AKW rate ratio
All UI	35.8	11.1	3.2*	13.3	2.7*	45.2	19.2	3.3*	14.4	3.1*	25.7	10.3	3.4*	7.7	3.3*
Drowning/submersion	5.1	1.1	4.7*	1.2	4.2*	8.3	1.6	5.4*	1.9	4.3*	<1.8	0.6	-	<0.6	**3.7**
Motor vehicle-traffic	7.9	5.8	**1.4**	4.4	1.8*	7.5	7.2	**1.0**	4.9	**1.5**	8.4	4.3	2.0*	4.0	2.1*
Transport-other (land)	3.2	0.3	10.8*	0.4	8.4*	4.6	0.4	11.1*	0.6	7.7*	<1.8	0.2	*	<0.6	>1*
Poisoning	3.2	1.1	2.9*	1.5	2.2*	3.3	1.6	2.0*	1.3	**2.5**	3.1	0.6	5.1*	1.4	**2.2**
Natural environment	2.6	0.1	20.0*	0.2	11.2*	4.6	0.2	28.1*	0.4	10.3*	<1.8	0.1	**NS**	0	**NS**
WISQARS ICD10 code groups 20–44 years															
All UI	120.8	42.0	2.9*	45.2	2.7*	159.6	57.0	2.9*	60.5	3.5*	79.4	19.5	3.0*	22.8	2.6*
Poisoning	54.0	20.1	2.7*	21.8	2.5*	58.0	27.6	2.1*	28.7	2.0*	49.9	12.5	4.0*	13.8	3.6*
Motor vehicle-traffic	17.6	15.5	**1.1**	9.4	1.9*	20.6	22.9	**0.9**	13.2	1.6*	14.5	7.9	1.8*	5.1	2.8*
Drowning/submersion	14.4	1.0	14.0*	2.6	5.6*	25.4	1.7	15.2*	4.0	6.4*	2.5	0.4	6.8*	1.0	**2.6**
Natural environment	9.5	0.3	33.6*	1.2	8.3*	13.9	0.4	31.9*	1.8	7.6*	5.0	0.1	39.7*	0.4	14.0*
Transport-other (land)	8.6	0.5	15.9*	2.1	4.1*	13.9	0.9	15.8*	3.0	4.7*	3.0	0.2	16.2*	1.1	**2.8**
WISQARS ICD10 code groups 45–64 years															
All UI	123.6	44.9	2.8*	51.5	2.4*	167.2	52.6	3.1*	61.6	2.7*	81.1	21.9	2.7*	28.6	2.8*
Poisoning	53.9	19.2	2.8*	21.4	2.5*	62.0	23.4	2.7*	27.0	2.3*	46.1	15.1	3.1*	15.1	3.1*
Motor vehicle-traffic	12.5	12.4	**1.0**	10.0	1.3*	20.4	18.3	**1.1**	13.0	1.6*	4.8	6.6	**0.7**	6.5	**0.7**
Natural environment	14.1	0.7	21.2*	1.8	7.8*	17.9	1.0	18.0*	2.7	6.8*	10.3	0.3	30.2*	0.9	12.0*
Transport-other (land)	4.0	0.5	7.4*	1.4	3.0*	6.5	0.9	7.3*	2.2	3.0*	<3.2	0.2	**NS**	<0.6	**NS**
Drowning/submersion	9.7	1.1	8.5*	3.0	3.2*	14.7	1.8	8.4*	5.1	2.9*	4.8	0.5	8.9*	0.7	6.6*
WISQARS ICD10 code groups 65+ years															
All UI	170.7	108.7	1.6*	98.2	1.7*	207.7	115.8	2.0*	123.2	1.7*	139.9	88.2	1.3*	97.4	1.4*
Motor vehicle-traffic	12.6	15.6	**0.8**	13.7	**0.9**	16.6	21.6	**0.8**	19.3	**0.9**	<9.2	11.0	**NS**	8.1	**NS**
Natural environment	16.3	1.4	11.7*	2.0	8.2*	19.4	1.8	10.9*	3.5	5.5*	13.8	1.1	12.5*	<1.8	>1*-
Drowning/submersion	7.5	1.3	5.8*	2.7	2.8*	16.6	2.1	7.9*	4.0	4.2*	0	0.7	**0**	<1.8	**NS**
Transport-other (land)	11.3	0.8	14.6*	0.4	25.5*	22.2	1.4	15.6*	0.9	25.2*	<9.2	0.3	**NS**	<1.8	**NS**
Poisoning	12.6	3.7	3.4*	6.4	2.0*	11.1	4.2	**2.6**	7.9	**1.4**	13.8	3.3	4.2*	4.9	2.8*

*Significantly different from 1, p< 0.05. NS: exact value cannot be reported in order to protect privacy, but rate ratio not significantly different from 1.

As shown in Table 4, the age-adjusted AN mortality rate for all UI among remote residents was significantly higher than for urban residents, which was significantly higher than the rural resident rate. A similar, though attenuated, pattern was exhibited among AKW. Age-adjusted mortality rate for all UI in remote and urban locations was significantly higher among AN people than among AKWs (RR = 2.4, 95% CI 1.7–3.5 and RR = 2.2, 95% CI 2.0–2.5 respectively). However, the overall UI rate was comparable between the 2 races (RR = 1.28, 95% CI 0.9–2.1) in rural locations.

**Table 4.** Age-adjusted unintentional injury mortality rates per 100,000 by residence in urban, rural and remote regions for AN and AKW 2006–2015.

		AN deaths	AN rate	AKW deaths	AKW rate	AN/AKW ratio
All UI	Urban	619	111.2	1821	49.9	2.2*
	Rural	44	51.1	291	39.9	1.3
	Remote	437	160.8	91	66.9	2.4*
Poisoning	Urban	280	46.8	671	16.0	2.9*
	Rural	10	11.4	77	9.1	1.3
	Remote	89	31.0	14	5.9	5.3*
Motor vehicle-traffic	Urban	88	13.8	333	8.4	1.6*
	Rural	14	16.0	92	12.0	1.3
	Remote	47	15.1	15	11.6	1.3
Drowning/submersion	Urban	39	6.5	92	2.4	2.8*
	Rural	8	8.7	16	2.0	4.3*
	Remote	64	21.1	11	6.0	3.5*
Natural environment	Urban	41	7.7	44	1.1	7.2*
	Rural	2	1.8	11	1.4	1.3
	Remote	56	19.7	5	3.5	5.6*
Transport-other	Urban	25	4.0	42	1.0	4.0*
	Rural	1	1.7	20	2.4	0.7
	Remote	43	14.9	3	1.5	9.9

*Significantly different from 1, p< 0.05.

*Poisoning* was the leading category of UI death among AN people overall, and in both males and females. As shown in Table 1, rates were nearly triple that of USWs and 2.5 times higher than AKWs. Stratified by sex, male AN poisoning deaths were more than double and female AN rates almost double the respective rates among USWs or AKWs, with the highest rates observed among urban residents and lowest among rural residents. While *poisoning* mortality was also highest among urban residents who were AKW, it was lowest among remote residents, as shown in Table 3. A large majority of poisoning deaths among both AN and AKW occurred in urban locations (more than 80% and more than 90%, respectively). Within poisoning, the greatest differences between AN and AKW or USW were alcohol poisonings (RR = 12.0, 95% CI 8.9–16.2 and 26.9, 95% CI 23.0–31.6 respectively). Differences were most amplified among AN females vs. AKW or USW females, a pattern also exhibited, but to a lesser extent, with *narcotics psychodyselptics [hallucinogens], not elsewhere classified*.

While AN mortality for UI involving *motor vehicle-traffic* incidents were 50% higher than among AKWs, they were similar to USWs (and similar between AN people classified as urban, rural or remote residents). Motor vehicle-traffic deaths among urban residents were 1.6 times higher among AN people than AKWs (95% CI 1.3–2.2), while rates among more isolated residents were not statistically different by race.

*Drowning/submersion* was the third leading cause of UI death among AN people. Although these deaths include bathtub – and swimming pool-related deaths, only 5% of AN and 19% of AKW deaths involved such circumstances; most drowning deaths occurred in natural waters or were unspecified. AN *drowning/submersion* mortality rates were higher across all age groups as compared to AKWs. The highest mortality was observed among AN males aged 20–44 years; rates were significantly higher among remote residents of both AN and AKW origin. Due to the important role of natural waters in AN subsistence activities, we examined death by *drowning/submersion* and *transport-other (water, air, space)* drowning. Combining both sources of drowning, AN people were more than 4 times as likely as AKWs to die from drowning. Boating-related drownings were the most frequent AN cause of death within the *transportation-other* category, differing from AKW, for whom deaths due to aircraft were most common in this category.

*Natural environment* mortality was the fourth leading cause of AN UI mortality. Significantly greater in AN people than both USW (RR = 20.7, 95% CI 16.6–26.0) and AKW (RR = 8.4, 95% CI 5.8–12.1), it was twice as common among AN males as AN females. As shown in Table 3, rates increased with age among both sexes and both racial groups (AN and AKW). The large majority (94%) of AN *natural environment* deaths were caused by exposure to cold; AKW deaths in this category included 55% due to cold exposure and 28% due to avalanches; all avalanche deaths were among AKW males; no such occurrences among AKW females, or AN of either sex. Most of the remaining few deaths were due to wildlife (primarily encounters with moose, bear, and wasp). *Natural environment* mortality rates among remote and urban AN were significantly different from remote and urban AKW rates, but similar between races in rural locations. Alcohol was listed as a factor in 21% of all AN *natural environment* deaths.

Death by *transport-other (land)* UI was the fifth leading cause of unintentional injury death among AN people. Most (94%) of non-traffic land vehicle deaths among AN people involved off-road vehicles. AN mortality in this category was greater than both USW (RR = 12.4, 95% CI 9.4–16.5) and AKW (RR = 5.1, 95% CI 3.4–7.4), as shown in Table 1. AN mortality was greater in males than females and the highest mortality rate occurred among males of age 65+. We identified an additional 67 AN and 30 AKW snow machining deaths attributed to other unintentional injury categories, including crashes, drowning following breaking through ice, freezing after getting lost and avalanches while snow machining. Mortality rates for snow machine UI among AN (6/100,000) were 10.5 times higher than among AKWs (95% CI 6.4–17.0). AN mortality rates were highest for the 20–44 age group, much higher among males than females (11.4 vs. 0.7 per 100,000), and highest for residents of remote regions. This age and sex pattern occurred in AKWs as well.

## Discussion

Although UI mortality rates were significantly higher for AN people than USW in 4 of the 5 leading UI categories for AN people, the differences were dramatically greater in the 3 categories where deaths were associated with Alaska’s extreme geography and climate: natural environment (RR = 20.7), transport-other (land) (RR = 12.4) and drowning/submersion (RR = 9.1). Together, these 3 categories constituted 25% of all AN UI deaths, but less than 5% of USW UI deaths. AKW rates for these categories were also elevated relative to USW (though to a lesser magnitude than AN), indicating increased mortality rates among all Alaskans compared to USW. Proportionately, nearly 30% of AN live in remote villages, whereas only 3% of AKW do. Thus, exposure to the environmental hazards common to remote village residence differs by races. Stratifying UI mortality by place of residence rather than race (that is combining AN and AKW results), we found that all residents of remote regions experienced significantly higher mortality from transport-other (land) and drowning/submersion than did urban or rural residents, whose rates were fairly similar to each other.

The overall risk of UI mortality increased with age for all 3 populations, although the age groups with the greatest risk for individual injury categories varied. Death rates for poisoning were much higher for AN ages 20–44 and 45–64 while persons aged 65+ had the highest UI death rates of transport-other (land) and natural environment incidents. The majority of elder AN natural environment deaths were cold-related, perhaps a result of decline in body response to temperature change with increasing age [12,13].

The statistics in our tables are based on decedent’s place of residence; however, we also examined place of occurrence for UI deaths. A large majority of UI deaths of urban residents occurred in urban locations for both AN people and AKWs (88% and 95% respectively). Patterns were different among rural and remote village residents, for whom a substantial proportion of UI deaths occurred in urban areas (30–58%). This difference may reflect a common practice of rural and remote residents visiting or spending some of the winter months in urban areas.

### Leading UI mortality categories

Although poisoning rates were more than double for AN people compared to USWs and AKWs, rates have increased dramatically among all of these populations over the last 30 years. A recent National Center for Health Statistics study found that 89% of US (all races) poisoning deaths were due to drugs and the increase in drug poisoning accounted for the recent overall increase in poisoning mortality [14]. Forty-six percent of AN poisoning deaths were attributed to alcohol and 47% to drugs (OTC, prescription and illegal) differing from AKW, where a much smaller proportion of deaths were attributed to alcohol than to drugs (10% and 86%, respectively). Importantly, the pattern differences in proportion of poisoning deaths do not indicate a reduced mortality rate from drugs among AN relative to AKW; the crude rates of mortality from drugs are quite similar in AN and AKW. Rather, the proportional differences result from higher rates of alcohol deaths observed among AN rather than lower rates of drug poisoning. Notably, while the poisoning mortality rate among AKW females is about half that of AKW males, poisoning mortality rates are equally high in AN males and females. AN poisoning fatality rates were lower in rural areas, but similar in urban and remote areas, although access to alcohol (46% of poisoning deaths among AN) in urban areas is often easier (urban areas permit the sale and consumption of alcohol, while several rural and remote Alaskan communities do not) [15]. Importantly, in a 2008 study, 72% of AN alcohol poisoning deaths state-wide occurred in communities with legal access to alcohol although only 48% of AN people live in these communities [16]. As was observed for all UI deaths, poisoning UI deaths of urban residents (both AN and AKW) almost all occur in urban areas. In contrast, many poisoning deaths among rural and remote residents also occur in urban areas away from home (about 30–60% varying with race and rural/remote residence). This pattern may result from the greater propensity of rural and remote residents to travel to urban areas rather than urban residents to spend time in rural/remote regions.

Although we found no significant difference in mortality from motor vehicle-traffic crashes/incidents between AN people and USW, mortality per mile driven is likely much higher among AN people. Nearly 40% of the AN population live in rural or remote Alaska areas with few roads. Snow machines and ATVs are often used for transport rather than motor vehicles. The annual per capita motor vehicle traffic miles driven by AN people are likely much lower than that of USW [17,18]. Additionally, studies by the State of Alaska Department of Transportation, as well as Behavioral Risk Factor Surveillance System data, report lower seat belt usage by AN people compared to both AKWs and USWs, particularly in rural areas, contributing to motor vehicle fatality [19].

Expectedly, AN mortality from natural environment was highest among remote residents. However, the proportion of these deaths with alcohol or drugs listed as a factor among rural and remote residents (14%) was less than half that of urban AN residents (32%). Alcohol or other drugs were a contributing factor for a much higher proportion of natural environment deaths among AN than AKWs, a pattern also documented in a national study of American Indian/Alaska Native and USW mortality [20]. Although commonly believed to warm the body, alcohol consumption has been demonstrated to lower core body temperature in cold weather conditions, thereby exacerbating hypothermia risk [12,13].

Almost all types of transport-other (land) deaths among AN people involved off-road vehicles, primarily snow machines and ATVs. Greater dependence on off-road vehicles in rural remote AN communities creates greater risk for other land transport fatalities among rural AN residents than AKW or urban AN residents [21]. Similarly, dependence on fish and marine mammals as primary food sources and reliance on commercial fishing for income requires substantial exposure to natural waters, which increases risk for boating and non-boating UI and drowning. In an earlier study, AN people state-wide had drowning fatality rates nearly 4 times that of non-Native Alaskans [22]. The combined boating/non-boating UI mortality from the current analysis shows the disparity has not changed over time; the 12.8/100,000 AN drowning death rate remains 4.2 times that of AKW.

### Limitations

Racial misclassification on death certificates is a possible cause of under-reporting of UI deaths among AN; analysis of record linkages between Indian Health Service records and vital statistics records indicates that racial misclassification in Alaska is likely around 7% [23]. The true risk of motor vehicle injuries per mile driven may be greatly underestimated for those who use motor vehicles, especially in non-urban Alaska, as many persons who do not (or rarely) use motor vehicles are included in the rate calculations. The true unintentional mortality risk associated with alcohol and drug consumption is difficult to accurately assess as well. While the poisoning category identifies UI deaths directly resulting from alcohol or drug poisoning, plausibly many other UI deaths result at least in part from alcohol or drug consumption. However, lack of accurate data prevents a means to ascribe these other UI deaths to that influence.

Unintentional injury deaths vary by the remoteness of residence, which is itself a marker of lifestyle differences. We classified census places into 3 levels of isolation (urban, rural and remote) based on ease of travel to levels of health care. This classification system is imperfect, as it only partially captures substantial differences in lifestyle. For example, there are several towns of 4,000–6,000 people located hundreds of miles from the road system and many of their residents rely on subsistence lifestyle using boat and snow machine. However, their residents do have local access to level II hospital care. Their subsistence lifestyle (and its hazards) is far different from that of an AN person living in suburban Anchorage, even though both places are classified as “urban”. Distinctions in UI mortality due to lifestyle differences associated with the unusually varied residences in Alaska are likely attenuated by the limitations of the residence designation schema chosen. Furthermore, the classification of residence is more fluid among AN than among USW. Many extended families maintain residence in both their “home” community and in an urban area (typically Anchorage or Fairbanks), with much flow between the two, depending upon season and needs. Thus, an urban resident is not necessarily always urban, or a remote resident always remote.

### Conclusion

This study reveals several of UI mortality areas to target for prevention or that need further investigation. Nearly one third of AN and AKW UI deaths occurred when the deceased had travelled away from his/her community type of residence, mostly from more rural/remote to more urban areas. More investigation is needed to determine if this is due to a lack of familiarity with different environments and circumstances or the amount of time rural and urban residents spend in the other geographic areas. Because of variation in common modes of transportation used across the state (and in contrast to USW), a modified overall transportation category and associated fatality rate, integrating snow machine, ATV, motor vehicle and boating might allow a more consistent comparison of the burden of transportation injuries in the state and in comparison to elsewhere. Focused research to identify all the ways in which alcohol/drugs may have contributed to a UI death would improve our understanding of UI and inform prevention efforts. Continued and enhanced efforts to encourage personal flotation device use, as well as swimming instruction in regions of the state relying on boating as transportation can help reduce drowning and boating UI deaths. Similar educational efforts on the use of helmets when riding snow machines or ATVs may help alleviate UI deaths, as the majority of snow machine deaths involve head and neck injuries. A 2006 study found snow machine helmet usage rates to be much lower in rural areas (urban 81%, rural areas 47%) [24]. Alaska residents, particularly AN residents and remote village residents face higher risks of UI mortality, often as a result of the difficult natural environment conditions in which they work, travel and recreate.

## Supplementary Material

Appendix_A_Supplemental_Table.docx
