# Supplementary material for: Disproportionately higher unintentional injury mortality among Alaska Native people, 2006–2015
Source: Int J Circumpolar Health. 2018 Jan 18;77(1):1422671. doi: 10.1080/22423982.2017.1422671 (PMC5774398; doi:10.1080/22423982.2017.1422671)
Supplement: Appendix_A_Supplemental_Table.docx [file ZICH_A_1422671_SM9271.docx]

Appendix A Supplemental Table

| **Unintentional Injury Category** | **ICD-10 Coding** |
| --- | --- |
| **All Unintentional Injury** | V01-X59, X85-Y86 |
| **Drowning/Submersion** | W65-W74 |
| **Fall** | W00-W19 |
| **Motor Vehicle-Traffic** | V02-V04(.1, .9),V09.2, V12-V14 (.3-.9), V19 (.4-.6), V20-V28 (.3-.9), V30-V39 (.4-.9), V40-V49 (.4-.9), V50-V59 (.4-.9), V60-V69 (.4-.9), V70-V79 (.4-.9), V80 (.3-.5), V81.1, V82.1, V83-V86 (.0-.3), V87 (.0-.8), V89.2 |
| **Natural Environment** | W42,W43,W53-W64,W92-W99,X20-X39,X51-X57 |
| **Poisoning** | X40-X49 |
| **Transport-other (land)** | V20-V28(.0-.2),V29(.0-.3),V30-V39(.0-.3),V40-V49(.0-.3),V50-V59(.0-.3),V60-V69(.0-.3),V70-V79(.0-.3),V80(.0-.2,.6-.9),V81-V82(.0,.2-.9),V83-V86(.4-.9),V87.9,V88(.0-.9),V89(.0,.1-.3,.9) |
| **Transport-Other (Water, Air, Space)** | V90-V99 |
